# Peloruside A is a microtubule-stabilizing agent with exceptional anti-migratory properties in human endothelial cells

**DOI:** 10.18632/oncoscience.169

**Published:** 2015-06-12

**Authors:** Anutosh Ganguly, Fernando Cabral, Hailing Yang, Kamala D. Patel

**Affiliations:** ^1^ Department of Microbiology Immunology and Infectious Diseases, University of Calgary; ^2^ Department of Integrative Biology and Pharmacology, University of Texas Medical School; ^3^ University of Texas, MD Anderson Cancer Center, Houston, Texas; ^4^ Department of Physiology and Pharmacology, University of Calgary

**Keywords:** microtubule dynamics, cell migration, endothelial cells, angiogenesis

## Abstract

Peloruside A is a novel antimitotic drug originally isolated from the marine sponge *Mycale hentschieli*. Previous studies showed that peloruside A stabilizes microtubules by binding to a site on tubulin distinct from paclitaxel, another microtubule stabilizing drug. Peloruside A blocks mitosis, but little is known about the effects on other cellular activities. Here we report that peloruside A is the most potent microtubule inhibitor yet tested for its ability to block endothelial cell migration. Quantitative analysis indicated that it inhibits microtubule dynamics and endothelial cell migration at 1/200^th^ of the concentration needed to inhibit cell division (the cytotoxic concentration), indicating that it could potentially have a large margin of safety when used to specifically target angiogenesis. By comparison, paclitaxel, a well-known cancer therapeutic drug, suppresses cell migration at 1/13^th^ of its cytotoxic concentration; and vinblastine suppresses cell migration at just slightly below its cytotoxic antimitotic concentration. Thus, different microtubule targeted drugs have varying relative potencies for inhibition of cell migration versus cell division. The results suggest that peloruside A may be an especially useful agent for anti-angiogenesis therapy and point to the likelihood that other antimitotic drugs might be found with an even larger potential margin of safety.

## INTRODUCTION

Microtubule inhibitors are among the most successful and widely used agents in cancer chemotherapy [1]. To maximize their effectiveness in treating tumors and minimize their toxicity to patients, it is important to understand each drug's mechanism of action as well as the mechanisms by which tumor cells develop resistance to its use. Currently, drugs are given in cycles of high toxic doses followed by drug-free periods in which bone marrow cells are allowed to recover. The goal is to kill as many tumor cells as possible, but success is limited by toxicity to the patient and by the ability of tumor cells to continue growing during the recovery period [2]. The initial growth of the tumor along with continued cell growth during recovery provide the opportunity for drug resistant cells to arise. This therapeutic strategy is based on the ability of the drug to kill cells by interfering with assembly of the mitotic spindle apparatus thereby leading to defects in chromosome segregation, mitotic progression, and cell division [3].

Microtubule inhibitors have also been reported to inhibit cell migration and angiogenesis that potentially could be used as the basis for new therapeutic strategies [4, 5], but the development of alternative strategies is hampered by the prevailing view that these drugs act primarily by directly interfering with cell proliferation [3, 6]. However, recent studies have demonstrated that these agents can affect cell behavior by at least two specific mechanisms. At low concentrations, the drugs suppress microtubule dynamics and effectively inhibit cell migration [7]. At higher concentrations, the drugs alter microtubule detachment frequency from spindle poles, interfere with assembly of the mitotic spindle apparatus, and block cell division [7, 8]. Although the mechanism by which microtubule inhibitors block cell proliferation has been heavily investigated, relatively little is known about how lower drug concentrations suppress microtubule dynamics and inhibit cell migration [9]. This information is crucial for designing alternative therapeutic strategies. For example, determining the mechanisms by which low drug concentrations control the migration of vascular endothelial cells and thereby inhibit angiogenesis may make it possible to use low concentrations of microtubule inhibitors to treat tumors without producing the significant toxicity associated with the drugs' ability to block mitosis. The effectiveness of such a strategy will require the identification of compounds that are likely to have a large margin of safety between the concentrations needed to inhibit angiogenesis versus cell division.

Recent studies from our group using human endothelial cells show that similar concentrations of vinblastine suppress endothelial cell migration and cell division (the cytotoxic concentration), making it a poor candidate for producing minimal toxicity when used to prevent angiogenesis [4]. In contrast, paclitaxel suppresses cell migration at only 1/10^th^ of its cytotoxic concentration, suggesting that paclitaxel should be a better drug for anti-angiogenic therapy than vinblastine [4]. In our new studies we searched for drugs with an even better potential margin of safety and report that peloruside A is such a drug.

## RESULTS

### Peloruside A causes spindle defects and blocks cells in mitosis

**Figure 1:**
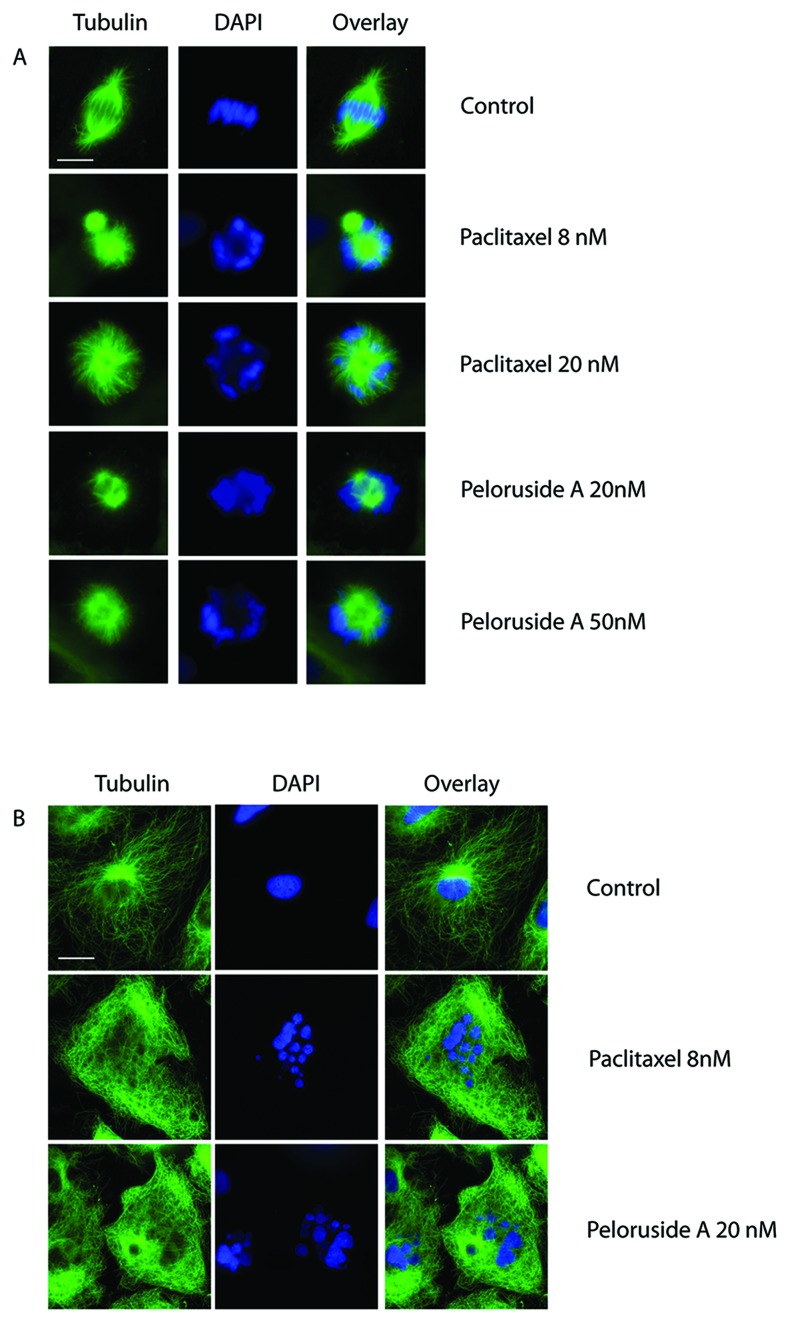
Drug effects on microtubule assembly and chromosome organization The IC_50_ for blocking mitosis is 8 nM for paclitaxel and 20 nM for peloruside A. HUVEC were treated with either paclitaxel or peloruside A for 48 h at either the IC_50_ or 2.5 times the IC_50_ concentration for blocking mitosis. The cells were then fixed and treated with tubulin antibodies to stain microtubules and with DAPI to stain the chromosomal DNA. Immunofluorescence microscopy of mitotic (A) and interphase (B) cells are shown. Scale bars are 5 μm in panel A and 10 μm in panel B.

**Figure 2:**
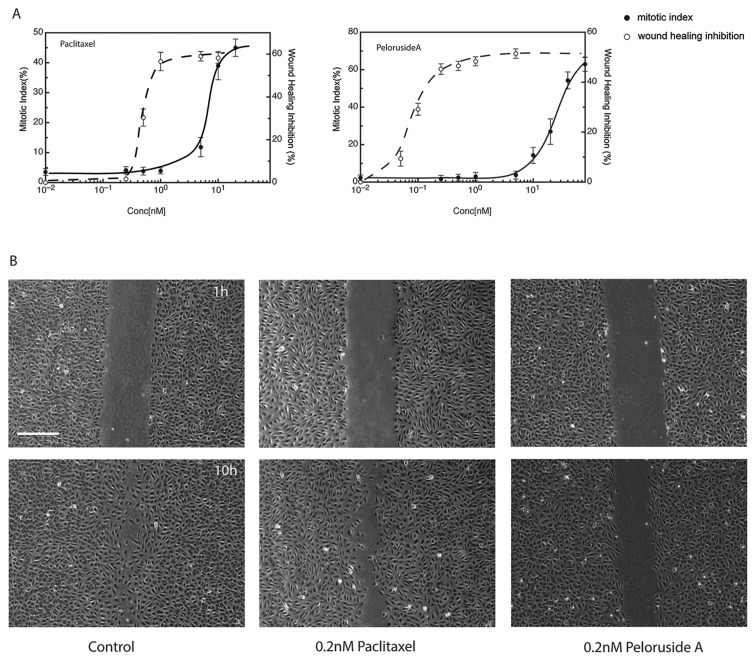
Drug effects on wound healing (A) Dose response curves for the inhibition of mitosis (measured as the mitotic index; i.e., the percentage of accumulated mitotic cells after 24 h) and the inhibition of wound healing after 10 h are shown in panel A for paclitaxel or peloruside A. For wound healing, a scratch wound was introduced into near confluent layers of HUVEC and the rate of wound closure was measured in the presence or absence of paclitaxel or peloruside A at the specified concentrations. (B) Panel B shows examples of the monolayers at 1 hour or 10 hours after introduction of the scratch under control conditions or at 0.2 nM of the specified drug. Scale bar in panel A is 100 μm.

Microtubule inhibitors impair spindle function and thereby block cells in mitosis. Like some of these other inhibitors, peloruside A has been found to bind tubulin, stabilize microtubules, interfere with mitotic spindle assembly, and arrest cell division in a number of cultured cell lines [10–12]. However, the effect of peloruside A on endothelial cells has not been examined even though information on the response of these cells is needed to evaluate the potential of the drug as an inhibitor of angiogenesis. To determine the cytotoxic antimitotic concentration, HUVEC were treated with different concentrations of peloruside A for 24 hours and the percentage of mitotic cells were counted to generate a dose response curve. Paclitaxel, whose effect on HUVEC was already known, was used as a positive control. As expected, peloruside A blocked cell division (Figure 1). The results indicated that paclitaxel was more potent in this assay and could inhibit mitosis at a concentration 2.5 times lower than peloruside A (IC_50_ value 8 nM versus 20 nM, Table 1). The mitotic index peaked at around 50% for both drugs (Figure 2A, solid line) and further increases in the drug concentrations did not produce a higher mitotic index. In addition to mitotic cells, we observed that about 20% of the cells could be stained with trypan blue and another 10% of the cells were multinucleated at the higher concentrations (data not shown). These data suggest that the percentage of mitotic cells that accumulated in the drug treated populations was limited by cell death and by slippage through the mitotic block into the next cell cycle without cell division [13].

**Table 1:** Comparison of Peloruside A and Paclitaxel for inhibition of different biological activities

Biological Activity	Paclitaxel (nM) (IC_50_)	Peloruside A (nM) (IC_50_)
Cell division	8.0	20.0
Cell migration	0.65	0.10
Microtubule dynamicity	0.50	0.10
Tube formation	0.50	0.10

To determine whether the effects on mitosis were accompanied by visible changes in the microtubule cytoskeleton, tubulin immunofluorescence was examined. At IC_50_ and higher concentrations, mitotic cells treated with peloruside A or paclitaxel exhibited multipolar and distorted spindles (Figure 1A). These spindle defects caused chromosomes to missegregate and prevented cells from dividing; and this, in turn, led to the appearance of multinucleated endothelial cells in subsequent cell cycles (Figure 1B). Interphase cells also exhibited obvious drug effects in the form of microtubule bundling at the cytotoxic drug concentrations. We conclude that at concentrations 20 nM and higher, peloruside A stabilizes and bundles microtubules, impairs mitotic spindle assembly, and blocks cell division in HUVEC.

### Peloruside A inhibits cell migration

We and others recently demonstrated that microtubule drugs can inhibit cell migration as well as cell division in cultured cell lines [7, 14, 15] but little is known on the effect of peloruside A on endothelial cell migration. To test whether peloruside A can inhibit endothelial cell migration, a wound healing experiment was carried out using human umbilical vein endothelial cells (HUVEC) at a series of drug concentrations using a scratch assay in a confluent monolayer. A comparison of peloruside A and paclitaxel for their effects on cell migration and mitosis is shown in Figure 2. The IC_50_ values for inhibition of cell migration calculated from Figure 2A were 0.1 nM for peluroside A and 0.65 nM for paclitaxel (Table 1). Example images of wound closure under control conditions or following treatment with 0.2 nM of either drug are shown in Figure 2B. Untreated endothelial cells migrated at 12.2 μm/h, a rate similar to previously published values. At this rate endothelial cells were able to almost completely close the gap in a wounded monolayer within 10 hours (Figure 2B-left panel). Cells treated with 0.2 nM peloruside A moved at a reduced rate of 4.8 mm/h and retained a sizable gap at 10 hours (Figure 2B-right panel). Notably, cells treated with the same concentration of paclitaxel experienced no significant decrease in cell migration (rate = 12.0 μm/h, Figure 2B-middle panel). The results showed that even though peloruside A is a weaker inhibitor of mitosis than paclitaxel, it is a more potent inhibitor of endothelial cell migration.

### Peloruside A inhibits microtubule dynamics at anti-migratory concentrations

**Figure 3:**
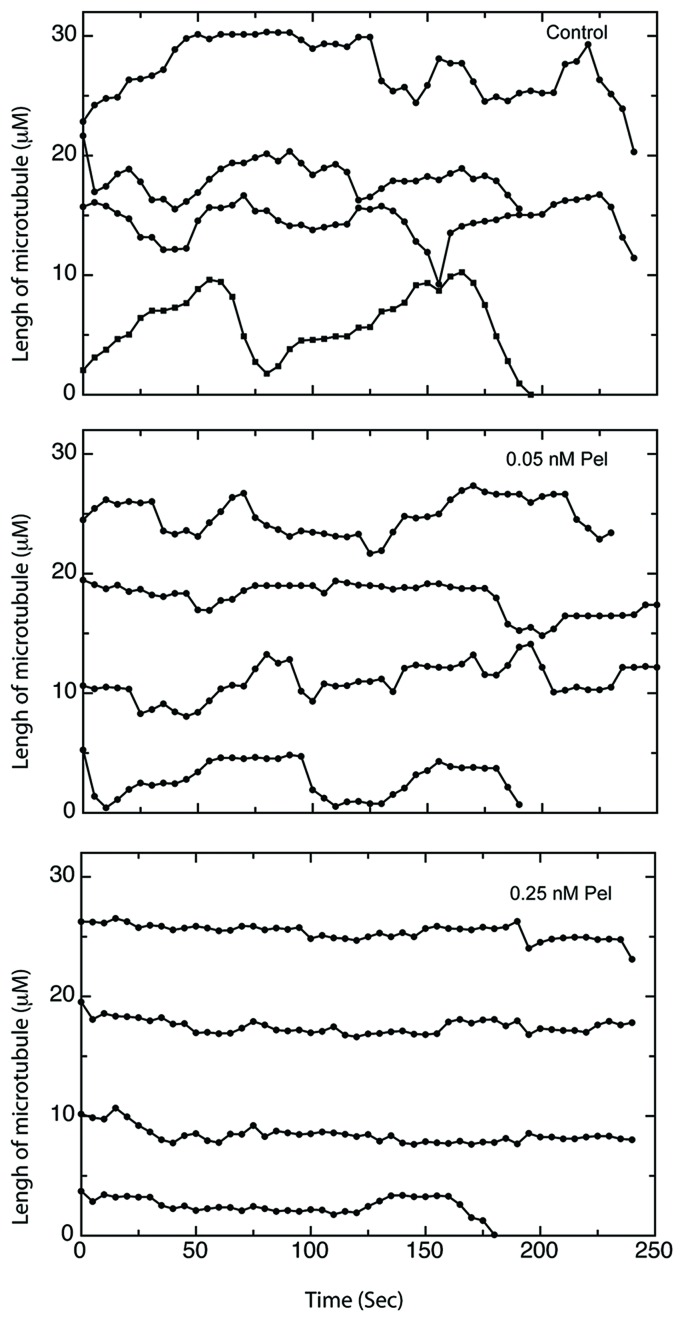
Life history plots of microtubules at anti-migratory concentrations of peloruside A HUVEC transfected with EGFP-MAP4 were treated with varying concentrations of peloruside A and microtubule lengths were measured as a function of time to generate life history plots. Traces for 4 separate microtubules are shown in each panel and their lengths at time zero were measured from arbitrary points that do not reflect their actual total lengths in the cell. Untreated microtubules (Control) were seen to grow and shorten repeatedly over the time course of the experiment. In contrast, microtubules treated with 0.05 nM peloruside A (0.05 nM Pel) exhibited fewer changes in length, and microtubules treated with 0.25 nM peloruside A (0.25 nM Pel) exhibited little to no changes in length.

Because peloruside A inhibited cell migration at only 1/200th of the antimitotic concentration, we questioned whether this inhibition was tied to suppression of microtubule dynamics or whether it might be acting by some other mechanism. To demonstrate the effect of peloruside A on dynamics, life-history plots of endothelial cell microtubules were generated to show time-dependent changes in length in the presence and absence of the drug (Figure 3). In the absence of peloruside A the endothelial cells exhibited typical stochastic growth and shortening episodes interspersed with periods of pause in which there was no significant change in length (Figure 3A). For the drug treatments, we chose three concentrations of peloruside A: 0.05 nM that had only a small effect on cell migration (a 14% decrease from 12.2 μm/h to 10.40 μm/h); 0.25 nM that produced a large effect on cell migration (a 60% decrease to 4.8 μm/h), but had no effect on mitosis (mitotic index 1.2); and 20 nM, the IC_50_ for mitosis. The lowest concentration that produced only a small effect on cell migration also had only a small effect on microtubule dynamicity (a decrease from 5.87 ± 0.43 to 5.07 ± 0.81 μm/min) (see Table 2). 0.25 nM peloruside A had little to no effect on mitosis (Figure 2A), but it decreased dynamicity by 60% (5.87 ± 0.43 to 2.32 ± 0.26 μm/min). This concentration also greatly increased the duration of pause for individual microtubules (from 47.3 ± 5.6 to 79.4 ± 2.4%) yet it had only small effects on the shortening rate (15.68 ± 1.22 to 13.15 ± 1.60 μm/min). The catastrophe frequency was reduced by 40%, (from 2.02 ± 0.21 to 1.21 ± 0.15) and there was a 31% increase in the rescue frequency (from 5.94 ± 0.59 to 7.78 ± 1.05). It should be noted that further increases in the peloruside A concentration did not further inhibit cell migration or further reduce dynamicity (from 2.32 ± 0.26 at 0.25 nM to 2.03 ± 0.34 at 20 nM concentration). We found a similar association between reduced microtubule dynamics and endothelial cell migration using paclitaxel [4]. This observation is consistent with our previous publications in this and other model systems [4, 14].

**Table 2:** Microtubule dynamics in HUVEC in the presence and absence of Peloruside A

Parameter	None	0.05 nM	0.25 nM	20 nM
Growth				
Rate (mm/min)	12.04 ± 0.75	12.73 ± 0.87	9.39 ± 0.49**	10.65 ± 1.4**
Duration (sec)	8.83 ± 0.65	7.71 ± 0.75	6.22 ± 1.10**	6.83 ± 1.06**
Distance (mm)	1.78 ± 0.17	1.65 ± 0.20	0.99 ± 0.19**	1.44 ± 0.48*
Shortening				
Rate (mm/min)	15.68 ± 1.22	17.26 ± 1.26	13.15 ± 1.60	12.13 ± 1.50
Duration (sec)	9.02 ± 0.42	8.50 ± 0.94	8.45 ± 1.21	6.18 ± 0.72**
Distance (mm)	2.50 ± 0.21	2.59 ± 0.42	1.90 ± 0.46**	1.23 ± 0.15**
Frequency				
Catastrophe (min^−1^)	2.02 ± 0.21	1.72 ± 0.13	1.21 ± 0.15**	0.97 ± 0.29**
Rescue (min^−1^)	5.94 ± 0.59	7.04 ± 0.82	7.78 ± 1.05*	9.7 ± 0.82**
Percentage time				
Growing (%)	19.5 ± 2.7	15.8 ± 2.2	7.6 ± 1.3**	6.7 ± 1.1**
Shortening (%)	21.5 ± 3.4	18.3 ± 1.5	13.0 ± 1.5*	10.2 ± 1.2**
Pausing (%)	47.3 ± 5.6	61.9 ± 4.8*	79.4 ± 2.4**	84.1 ± 1.8**
Dynamicity (mm/min)	5.87 ± 0.43	5.07 ± 0.81	2.32 ± 0.26**	2.03 ± 0.34**
Microtubules counted	35	20	20	20

Values represent the mean±standard error. Catastrophe: the transition from either growth or pause phase to shortening phase. Rescue: the transition from shortening phase to either growth or pause phase. Dynamicity: the total length change of individual microtubule during life history.

* *p* < 0.05

** *p* < 0.01 compared to cells at 0 concentration of peloruside A

### Peloruside A inhibits capillary tube formation

Capillary tube formation is an assay that is widely used to model the reorganization stage of angiogenesis by measuring the ability of endothelial cells, plated at sub-confluent densities with the appropriate extracellular matrix support, to form capillary-like structures. This approach is typically employed as a first step to determine the potential of various compounds to promote or inhibit angiogenesis[16]. Upon plating, the endothelial cells attach to the matrix and generate mechanical forces on the surrounding extracellular support to create tracks or guidance pathways that facilitate cellular migration [17]. The resulting cords of cells eventually form hollow lumens. Compounds that are able to inhibit tube formation could be useful in diseases such as cancer where delivery of oxygen and nutrients to tumor cells requires the formation of new blood vessels.

**Figure 4:**
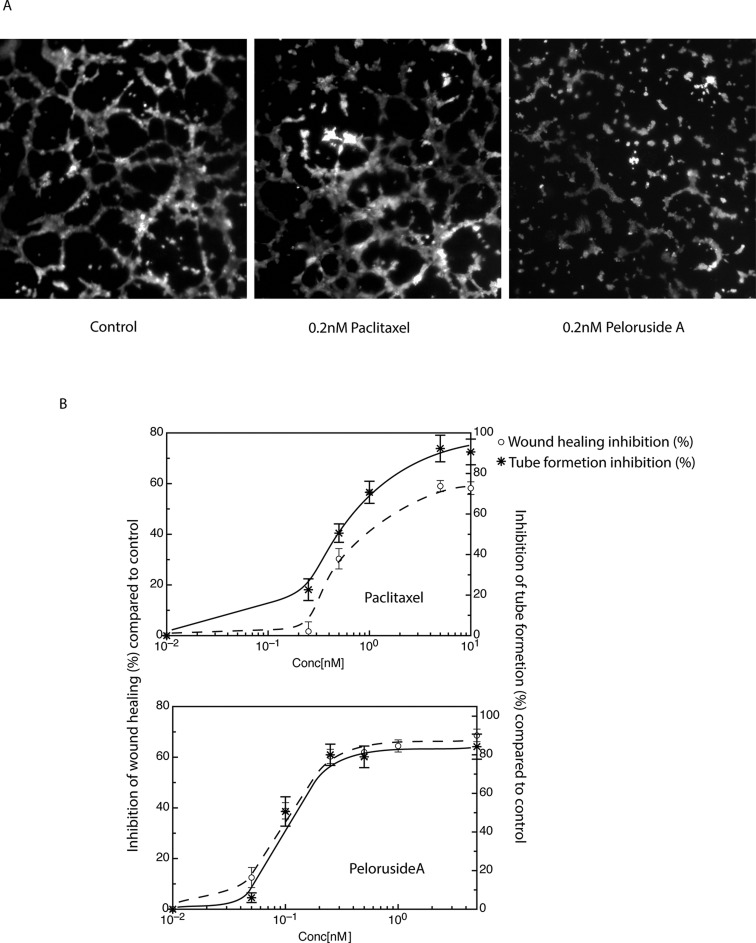
Comparison of the abilities of paclitaxel and peloruside A to inhibit capillary tube formation Endothelial cells (HUVEC) were plated at equal densities onto Matrigel in the presence and absence of the specified concentrations of paclitaxel or peloruside A. Cells were then examined after 5 h for the ability to form tubes. (A) Images of capillary tube formation at a single concentration of each drug are shown in panel A. (B) Dose response curves showing the inhibition of capillary tube formation as compared to wound healing is shown in panel B.

We tested the ability of endothelial cells to form these tube-like structures in the presence or absence of paclitaxel and peloruside A. For quantitation, the total tube length from 8 non-overlapping random microscopic fields was measured at each drug concentration and the experiment was repeated at least three times. Examples of some of these microscopic fields are shown in Figure 4A. Without any drug treatment endothelial cells formed a branched tube-like network. The total tube length in an average field of view was calculated to be 1406.8 ± 83.7 μm (Figure 4A, left panel). Treatment of HUVEC with 0.2 nM of peloruside A (twice the anti-migratory IC50 concentration) decreased the total tube length to 296.6 ± 73.0 μm, an 80% decrease. Treatment with 0.2 nM of paclitaxel reduced the total tube length to 1087 ± 91.1 μm, only a 20% decrease (Figure 4A, middle panel).

To determine whether inhibition of cell migration is the primary cause for the anti-angiogenic effect of peloruside A, inhibition of wound healing activity was compared to inhibition of tube formation at different concentrations of the drug (Figure 4B). The results indicated that the IC50 values for tube formation and wound healing were virtually identical for peloruside A (0.1 nM), and that paclitaxel was only marginally less potent in preventing cell migration (IC_50_ = 0.65 nM) compared to its antiangiogenic concentration (IC_50_ = 0.5 nM). Overall the data strongly suggest that the antiangiogenic activities of these two drugs are likely to be due to their anti-migratory effects although the possibility of other actions of the drugs cannot be ruled out.

## DISCUSSION

Microtubule inhibitors like paclitaxel, epothilone B and vinblastine are important drugs commonly used to treat patients with breast, lung, bladder, prostate, ovarian, melanoma, as well as other types of solid tumor cancers [18]. However, successful treatment is limited by the ability of tumor cells to gain resistance to chemotherapeutic agents resulting in relapse of the disease for a variety of reasons including overexpression of P-glycoprotein [19], mutations in tubulin that alter microtubule assembly [20–22] and altered tubulin composition resulting from changes in the production of tubulin isotypes [23] and microtubule associated/interacting proteins [24, 25]. Despite these limitations, microtubule inhibitors have been used for more than 50 years. Early work suggested that their primary mechanism of action was blocking cell division by interfering with the assembly and function of the mitotic spindle apparatus [3]. As a result cells treated with microtubule inhibitors were arrested in mitosis and died as a result of apoptosis. However, later studies showed that these drugs also inhibit the migration of cells in culture and produce *in vivo* anti-angiogenic actions that could contribute to their effectiveness as chemotherapeutic agents [4, 5]. For example, a number of studies during the last decade showed that paclitaxel has anti-angiogenic activity, as judged by its ability to inhibit either tubule formation or cell migration, as well as an anti-proliferative effect towards endothelial cells [5].

More recently, it has been proposed that microtubule inhibitors produce concentration-dependent effects on cell migration and cell division through distinct mechanisms. Microtubules are cylindrical polymers of aβ-tubulin heterodimers that extend from the centrosome, an organelle located near the cell nucleus, to the cell periphery. These structures exhibit dynamic instability, a behavior that is characterized by intermittent episodes of growth and shrinkage interrupted by periods of rest or “pause.” Low concentrations of microtubule inhibitors like paclitaxel, colcemid, and vinblastine suppress microtubule dynamics and concomitantly block cell migration by preventing remodeling of microtubules in the migrating cells [14]. In contrast, inhibition of cell division generally requires higher drug concentrations [7, 14]. At these higher concentrations, microtubule inhibitors act by affecting the ability of microtubules to remain attached to the centrosome [7, 8, 26]. As a result, mitotic spindle assembly is inhibited, chromosome segregation is disrupted, cell cycle progression is blocked, and cells either die quickly by apoptosis or they slip through the mitotic block as multiploid undivided cells that die at a later time [7, 8]. A recent study using primary endothelial cells further showed that the relative concentrations needed to inhibit cell migration versus cell division are drug-specific [4]. For example, vinblastine inhibited cell migration and microtubule dynamics at a concentration that was only slightly below the concentration that inhibited mitosis [4]. In contrast, paclitaxel inhibited cell migration and dynamics at least 10X fold lower than the antimitotic concentration [4]. These results suggested that differences in the potencies of drugs to inhibit cell migration versus cell division could be exploited to devise therapies specifically targeted to interfere with angiogenesis.

Based on these findings, we explored novel drugs in an attempt to identify those with superior ability to inhibit cell migration and angiogenesis. Peloruside A is a microtubule inhibitor isolated from marine sponges [10] that hyperstabilizes microtubules in a manner similar to paclitaxel, even though it binds to a separate site. As a potential chemotherapeutic agent, peloruside A offers several advantages over paclitaxel. For example, it is not a substrate for P-glycoprotein [27] and its availability may be better following its recent chemical synthesis [28]. The optimal clinical use of this drug, however, will require a detailed understanding of its mechanism of action. In this study we report that peloruside A is a very effective and potent agent in its ability to suppress microtubule dynamics and inhibit endothelial cell migration, and that it is able to elicit these effects at a concentration that is 200 times lower than the concentration needed to inhibit cell division. The low concentration that inhibited cell migration was also shown to similarly inhibit capillary tube formation, a predictor of anti-angiogenic activity. Because most of the toxic side effects of antimitotic drugs arise from inhibition of mitosis and cell division, our results suggest that peloruside A should be a particularly safe and effective drug for use as an anti-angiogenesis agent. Compared to paclitaxel, a drug that we previously reported to have good separation between antimigratory and antimitotic concentrations, peloruside A is both a weaker antimitotic drug that is less prone to elicit toxic side effects, as well as a more potent antimigratory drug predicted to have stronger anti-angiogenic activity.

Given the wide gulf between potential anti-angiogenic activity and toxic antimitotic concentrations for peloruside A, we envision the possibility that the drug could be given at very low non-toxic concentrations on a continual basis to maintain patients in remission by preventing the growth of any residual small tumors that were not completely eliminated by induction chemotherapy. Because of the drug's ability to inhibit cell migration, we also envision the possibility that similar low drug doses will also suppress the ability of cancer cells to leave the primary tumor and metastasize to other sites. The powerful effects of peloruside A on the movement of cells in culture make this drug a strong candidate for further study and development.

## MATERIALS AND METHODS

### Materials

Peloruside A was a generous gift from Dr. David Schrimer, University of Calgary. Monoclonal antibody DM1A to α-tubulin was purchased from Sigma-Aldrich. Alexa-conjugated goat anti-mouse IgG and Calcein were purchased from Invitrogen. Matrigel was purchased from BD Biosciences. JetPEI-HUVEC was purchased from Polyplus. All other chemicals were purchased from either Sigma-Aldrich or Fisher Scientific.

### Isolation of human vascular endothelial cells

Human umbilical vein endothelial cells were isolated and maintained as previously demonstrated [29]. Briefly, endothelial cells were isolated from human umbilical cords (Foothills Hospital, Calgary, Canada). They were grown on a matrix of 0.2% gelatin and maintained in M199 with 20% human serum. Cells from the first passage were used in all experiments.

### Mitotic index

Endothelial cells were grown for 24 hours on coverslips coated with 2% gelatin and 25 μM fibronectin before being treated with microtubule inhibitors for a further 24 hours. The cells were fixed in 2% paraformaldehyde in PBS for 10 min and then permeabilized in PBS containing 0.5% Triton X-100. The permeabilized cells were stained with DAPI for a period of 30 min, washed in HBSS for another 30 min, and then observed under a fluorescence microscope with a 40X objective. The cells in mitosis were identified by their condensed chromosomes.

### Immunofluorescence

Endothelial cells were grown to 50-70% confluence on coverslips coated with 2% gelatin and 25 μM fibronectin, washed in PBS, and fixed in methanol at −20°C. To minimize background, the cells were pre-extracted before fixation with microtubule stabilizing buffer containing 20 mM Tris-HCl (pH 6.8), 1mM MgCl_2_, 2mM EGTA and 0.5% Nonidet P40 at 4°C for one min. They were then stained with a monoclonal mouse antibody to a-tubulin for 1 hour. The coverslips were washed and the cells were then counterstained with Alexa 488-conjugated goat anti-mouse IgG that included 1 μg/ml DAPI. After a final wash the coverslips were mounted on slides with Prolong Gold mounting media. Images were taken using a wide field microscope (Olympus) with a CCD camera and Velocity software.

### Cell migration assay

Endothelial cells were grown on plastic bottom 35 mm dishes coated with gelatin and fibronectin until they reached confluence. A scratch was introduced in the middle of the monolayer and the cells were then treated with different concentrations of peloruside A. The width of the scratch was measured immediately after addition of peloruside A and 10 hours after the cells had been allowed to migrate into the wound. The wound-healing rate was measured by calculating the distance between the edges of wound at 10 separate locations after 10 hours of incubation. The migration rate was calculated by dividing half the change in the distance between edges of the wound divided by time.

### Capillary tube formation assay

Endothelial cells were plated on ibidi-bottom angiogenesis μ-slides coated with 10 μl of matrigel. Endothelial cells were plated at 80% of confluency in the presence or absence of peloruside A or paclitaxel. After 5 hours, 2 μg/ml calcein was added to fluorescently label the cells and incubation was continued for 30 minutes. Fresh media was then added and imaging was carried out using a 4X or 10X objective.

### Live cell imaging

Endothelial cells were plated on ibidi bottomed 35 mm dishes and then transfected with GFP-MAP4 using JetPEI-HUVEC transfection agent (Polyplus transfection TM). At 24 hours following transfection, the cells were transferred to phenol red free medium and images were captured using a 100X 1.35 NA objective on a wide-field imaging system (DeltaVision, Applied Precision) with the SoftworX software supplied by the vendor. Images were taken every 5 s for 50 frames.

### Calculation of microtubule dynamics

Microtubule dynamics were calculated using ImageJ software using a method described in earlier publications [4].

### Statistics

All experiments were performed at least three times. The data were analyzed using a t-test when comparing two groups and analysis of variance with the appropriate post-tests when comparing more than two groups. Nonparametric tests were used when required. P < 0.05 was considered significant.
